# Congenital Absence of the Left Atrial Appendage and Possible Effects on Thrombosis: A Case Report

**DOI:** 10.7759/cureus.85333

**Published:** 2025-06-04

**Authors:** Harthik Kambhampati, Shivang Patel, Matthew Thomas, Jalal Ibrahim, Ambar Patel

**Affiliations:** 1 Medicine, Lake Erie College of Osteopathic Medicine, Bradenton, USA; 2 Pediatrics and Child Health, Lake Erie College of Osteopathic Medicine, Bradenton, USA; 3 Cardiology, St. Vincent's Medical Center, Jacksonville, USA

**Keywords:** cardiology, case report, left atrial appendage absence, radiology, transthoracic echocardiography

## Abstract

The left atrial appendage (LAA) is a common site for thrombus formation in atrial fibrillation (AF) and often the focus of imaging prior to cardioversion. We present a rare case of a 64-year-old male undergoing routine transesophageal echocardiography (TEE) prior to electrical cardioversion, during which the LAA was not visualized. Further evaluation confirmed congenital absence of the LAA - a seldom-reported finding with uncertain implications for stroke risk and anticoagulation management. This report adds to the limited literature on this anomaly and underscores the importance of a systematic approach when faced with unexpected imaging findings. Although the absence of the LAA might theoretically reduce thromboembolic risk, current evidence is insufficient to alter management, and standard anticoagulation guidelines for AF should still be followed.

## Introduction

In patients with atrial fibrillation, the left atrial appendage (LAA) is a key anatomical region due to its propensity for harboring thrombi. It accounts for approximately 90% of atrial thrombi in non-rheumatic AF and around 60% in rheumatic mitral valve disease [1]. The LAA is also the target for various occlusion strategies aimed at reducing stroke risk. Because some patients have contraindications to anticoagulation, LAA occlusion has become an important alternative for thromboembolism prophylaxis [1]. Its absence, particularly as a congenital anomaly, is extraordinarily rare. This unique anatomical variant may theoretically reduce thromboembolic risk, but its clinical implications for anticoagulation decisions remain uncertain. In this report, we describe an incidental discovery of LAA agenesis during pre-procedural imaging in a patient scheduled for cardioversion and discuss its clinical implications and diagnostic approach.

The LAA is a pouch-like extension of the left atrium located within the pericardium near the left ventricle [1]. It functions as a decompression chamber during left ventricular systole, a role attributed to its elevated position in the atrium, greater distensibility, high levels of atrial natriuretic factor (ANF), and distinct neural innervation [1].

The LAA’s trabeculated interior and narrow neck contribute to blood stasis, making it the most common site for thrombus formation in atrial fibrillation. These anatomical characteristics also underlie its diagnostic significance, as failure to visualize the LAA on imaging can raise suspicion for thrombus, prior surgical exclusion, or device occlusion.

Here, we present the case of a 64-year-old man with paroxysmal atrial fibrillation who was incidentally found to have a congenital absence of the LAA during routine pre-cardioversion TEE. Given that the LAA is a primary site of thrombus formation in atrial fibrillation, this rare finding highlights the importance of systematic imaging review and raises questions about stroke risk and anticoagulation strategies in the absence of this anatomical structure.

## Case presentation

**Figure 1:**
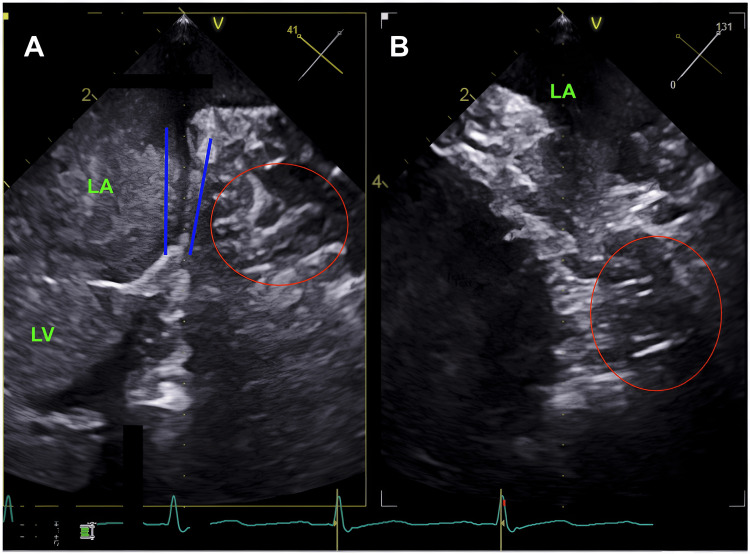
Transesophageal echocardiogram (TEE) of left atrium at 41° and 131° oblique angles LA = Left atrium, LV = Left ventricle, red circle = Congenital absence of left atrial appendage (LAA). (A) 41° oblique image shows contrast (gray behind the "LA" and "LV") filling the left atrium and left ventricle. The blue lines outline the sharp demarcation of where the LAA should connect with the LA, and the area next to the LA shows no filling within the theoretical space of the LAA, supportive of congenital absence. The red circle outlines this hypoechoic chamber, which is anomalous in relation to where the LAA should be. (B) 131° oblique image shows another angle confirming a hypoechoic structure where the left atrial appendage should be, which is highlighted by the red circle.

A 64-year-old man with paroxysmal atrial fibrillation was referred for elective electrical cardioversion after suboptimal response to rate-control therapy. He had no history of cardiac surgery, device implantation, or structural heart disease. As per protocol, a TEE was performed to exclude intracardiac thrombus. Despite multiple probe manipulations and standard oblique views (0°, 45°, 90°, 135°), the LAA could not be visualized. Instead, a small hypoechoic area was noted in the expected region of the LAA with no evidence of echo-dense material. It lacked trabeculations, surgical ligation, previous scarring indicating thrombosis, or Doppler flow, suggesting that it was neither a rudimentary appendage nor an artifact (Figure 1). Additionally, the TEE Figure further shows the lack of CT contrast filling the LAA where the arrow is pointing, which alerted the physician about a possible congenital LAA at the time (Figure 1).

**Figure 2:**
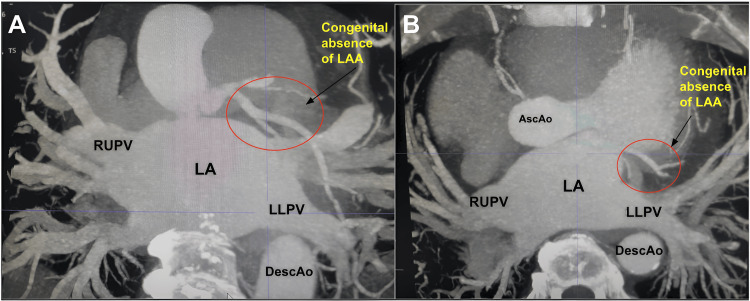
Confirmational cardiac CT scan RUPV = Right upper pulmonary vein, LA = Left atrium, LLPV = Left lower pulmonary vein, DescAo = Descending aorta, AscAo = Ascending aorta, LAA = Left atrial appendage (A) The red circle showing congenital absence of LAA. The cardiac CT scan was done to confirm the results of the TEE, and it shows no continuity of the left atrial appendage with the left atrium supporting the diagnosis of congenital absence of left atrial appendage. (B) Secondary angle view supporting the congenital absence of LAA outlined by the red circle.

These findings raised suspicion for congenital absence, prompting further evaluation with cardiac CT. CT angiography confirmed complete absence of the LAA, with no remnant stump, surgical scar, or occlusion device-distinguishing it from acquired causes. CT provided definitive confirmation by demonstrating no anatomical continuity between the left atrium and any outpouching in the expected location of the LAA (Figure 2).

The patient had a CHA₂DS₂-VASc score of 3, indicating moderate stroke risk. Although the absence of an LAA might suggest a lower embolic risk due to a lack of thrombus-prone anatomy, the limited data on outcomes in such cases precludes deviation from established management. Therefore, anticoagulation with apixaban was continued per current AF guidelines and was recommended to undergo catheter ablation as a definitive rhythm control strategy to reduce potential complications and enhance long-term rhythm stability.

In the absence of contraindications, the patient initially underwent electrical cardioversion, which briefly restored sinus rhythm but was not sustained. He was subsequently referred to electrophysiology and successfully underwent catheter ablation, achieving durable rhythm control. At follow-up, he remained in sinus rhythm without complications. The diagnosis of congenital LAA absence was confirmed through concordant findings on both TEE and CT.

## Discussion

The absence of the LAA is an extremely rare anomaly with only 23 cases as of December 2024 reported in scientific literature [2]. The LAA originates during the third week of gestation as a remnant of the embryonic left atrium, distinct from the pulmonary vein-derived portions of the atrium. It develops from the fusion of paired cardiac mesoderm and forms a structurally and functionally unique chamber by the fourth to sixth week of embryogenesis [1].

Advanced imaging techniques, including TEEs, have made LAA visualization possible in most cases. However, failure to visualize the LAA should raise suspicion for possibilities such as complete thrombotic occlusion, prior surgical or percutaneous ligation, artifacts, suboptimal echocardiographic windows, or, as in our case, congenital absence [3]. Key distinguishing features include the absence of echo-dense material (suggestive of thrombus), the lack of scar tissue or remnant devices (suggesting no prior occlusion), and consistent findings across multiple imaging planes. In such scenarios, advanced imaging modalities such as multidetector CT and cardiac MRI can aid in accurate diagnosis and differentiation. Identifying congenital anatomical anomalies, as seen in this patient, is important and should be confirmed before proceeding with direct current (DC) cardioversion in patients with atrial fibrillation [3].

Although typically considered a benign structure, the LAA can become a site of clot formation in patients with atrial fibrillation due to reduced blood flow. These thrombi can embolize to systemic organs such as the brain or kidneys, leading to ischemic complications. Stroke prevention in atrial fibrillation largely centers on anticoagulation, guided by the CHA₂DS₂-VASc score, which helps approximate one's stroke risk. Direct oral anticoagulants (DOACs) or warfarin are commonly used to prevent clot formation and have been shown to reduce the risk of stroke by nearly two-thirds [4]. However, in patients who are unable to tolerate long-term anticoagulation, alternative strategies include left atrial appendage occlusion devices, such as the Watchman device or Amulet device, which physically seal off the LAA to prevent thrombus formation and embolization [5,6]. Both the Watchman and Amulet devices aim to close the LAA to reduce stroke risk in AF patients by removing the anatomical space where clot formation is thought to occur. Given that these procedures aim to eliminate the LAA as a source of thrombus, congenital absence may theoretically reduce stroke risk. However, this assumption lacks supporting data and should not currently influence management decisions.

Although clot risk may be theoretically reduced, careful consideration must be given to AF management in patients with congenital LAA. For example, in this case, catheter ablation was pursued as a definitive rhythm control strategy. However, the absence of the LAA could affect the success of catheter ablation, as it may alter left atrial anatomy and eliminate a potential arrhythmogenic site. While some data suggest the LAA can contribute to AF triggers, specific impacts of its congenital absence on ablation outcomes remain speculative. As a result, a customized approach to ablation may be needed to accommodate the patient’s unique heart anatomy [2].

Though the LAA is the most common site for thrombus formation in AF, stroke risk is multifactorial, influenced by age, sex, race/ethnicity, hypertension, smoking, diet, and physical inactivity. Current guidelines for managing stroke risk in AF patients incorporate these clinical factors through tools such as the CHA₂DS₂-VASc score but do not account for anatomical variations such as congenital absence of the LAA [7]. In this case, the patient's CHA₂DS₂-VASc score was 3, justifying continued anticoagulation despite presumed lower anatomic risk. There are no established recommendations for how such structural anomalies should influence anticoagulation decisions, leaving clinicians without clear guidance in these rare and complex scenarios. Consequently, clinical judgment becomes paramount. In the absence of evidence-based protocols, multidisciplinary discussion, imaging confirmation, and individualized decision-making are advisable to ensure safe and appropriate care.

In light of these uncertainties, ongoing research and case reporting are crucial to guide future recommendations and ensure safe, evidence-based management for patients with rare anatomical variants such as congenital absence of the LAA.

## Conclusions

This case illustrates an exceedingly rare anatomical variant found incidentally during routine pre-cardioversion imaging. Although the long-term clinical impact of LAA agenesis is not well-defined, recognition of this condition is essential to avoid misdiagnosis, such as mistaking it for thrombus, surgical ligation, or occlusion device placement, especially on transesophageal echocardiography, where hypoechoic or absent structures may lead to false assumptions. Multimodality imaging, particularly cardiac CT or MRI, plays a critical role in confirming the diagnosis and ruling out more common acquired conditions. In AF patients, standard anticoagulation strategies remain appropriate until more is understood about this anomaly’s role in stroke risk. The congenital absence of the LAA should also be identified preoperatively, as it may impact intraoperative strategy - such as the feasibility of LAA occlusion, mapping targets during ablation, or interpretation of surgical anatomy during cardiothoracic procedures. Given the lack of specific guidelines, management should align with established AF protocols for this patient and be guided by individualized risk assessment.

## References

[1] Sulague RM, Whitham T, Danganan LM, Effiom V, Candelario K, Latif N, Hameed I (2023). The left atrial appendage and atrial fibrillation—a contemporary review. J Clin Med.

[2] Batista Lage JG, Rangel RA, de Oliveira Júnior NA, Tavares Pinheiro MV, de Souza OF (2024). Congenital absence of the left atrial appendage: a positive coincidence for the electrophysiologist? A case report. Eur Heart J Case Rep.

[3] Ghori MA, Alessandro S (2015). Congenital absence of left atrial appendage: a case report and literature review. J Saudi Heart Assoc.

[4] D'Souza A, Butcher KS, Buck BH (2018). The multiple causes of stroke in atrial fibrillation: thinking broadly. Can J Cardiol.

[5] Beshai R (2022). Watchman device procedure complicated by rare perclose ProGlide embolization: case report and literature review. Cureus.

[6] Lakkireddy D, Thaler D, Ellis CR (2021). Amplatzer Amulet left atrial appendage occluder versus Watchman device for stroke prophylaxis (Amulet IDE): a randomized, controlled trial. Circulation.

[7] Boehme AK, Esenwa C, Elkind MS (2017). Stroke risk factors, genetics, and prevention. Circ Res.

